# Baseline and On-Treatment Absolute Lymphocyte Count-Defined Lymphopenia in Solid Tumors Treated With Immune Checkpoint Inhibitors: A Systematic Review With Structured Qualitative Synthesis

**DOI:** 10.7759/cureus.111111

**Published:** 2026-06-18

**Authors:** Badr Cherradi, Matthieu Pallandre, Tiphaine Gil, Wafaa Kaikani, Imad Barjij

**Affiliations:** 1 Medical Oncology, Moulay Youssef Regional Hospital Center, Casablanca, MAR; 2 Radiation Oncology, Institut de Cancérologie et Hématologie Universitaire, Saint Etienne, FRA; 3 Gastroenterology and Hepatology, University Hospital of Saint-Etienne, Saint Etienne, FRA; 4 Medical Oncology, Cheikh Khalifa International University Hospital, Mohammed VI University of Health Sciences (UM6SS), Casablanca, MAR; 5 Medical Oncology, National Institute of Oncology, Ibn Sina University Hospital, Rabat, MAR; 6 Faculty of Medicine and Pharmacy, Mohammed V University of Rabat, Rabat, MAR

**Keywords:** absolute lymphocyte count, immune checkpoint inhibitors, lymphopenia, prognosis, systematic review

## Abstract

Immune checkpoint inhibitors are widely used in solid tumors, but patient outcomes remain variable, and practical prognostic markers are still being clarified. This systematic review examined whether lymphopenia, defined by absolute lymphocyte count, assessed before treatment and/or during therapy, is associated with survival outcomes in adults with solid tumors treated with immune checkpoint inhibitors. A total of 21 studies were included, with sample sizes ranging from 22 to 18,186 patients. Several studies reported associations between lower absolute lymphocyte count and less favorable survival outcomes, although findings were heterogeneous and context-dependent. Overall, the available evidence suggests that absolute lymphocyte count-defined lymphopenia may have prognostic relevance in some settings, although substantial heterogeneity across studies limits firm conclusions and highlights the need for more standardized research.

## Introduction and background

Immune checkpoint inhibitors (ICIs) targeting programmed cell death protein 1 (PD-1), programmed death ligand 1 (PD-L1), and cytotoxic T-lymphocyte antigen 4 (CTLA-4) are now part of standard management across multiple solid tumors [1-3]. They are used as monotherapy, combined checkpoint blockade, or within broader systemic regimens. Despite their established role, outcomes remain heterogeneous. In routine practice and observational cohorts, patients differ in disease burden, prior anticancer therapies, performance status, comorbidities, and concomitant medications, all of which may influence prognosis and complicate cross-study comparisons [2,4]. This has increased interest in pragmatic prognostic factors that can be measured from standard clinical data and used for adjustment or stratification in time-to-event analyses such as overall survival (OS) and progression-free survival (PFS) [2,5,6].

Peripheral blood parameters are among the most accessible candidates for prognostic assessment in immuno-oncology. The absolute lymphocyte count (ALC), obtained from routine complete blood counts, provides a quantitative measure of circulating lymphocytes at a given timepoint and can be assessed both before ICI initiation and repeatedly during treatment [2,5]. Lymphocytes, especially T-cell populations, contribute to tumor-immune surveillance and are central to the intended mechanism of checkpoint blockade, which aims to restore or amplify antitumor immune activity [3,7]. ALC is therefore clinically attractive compared with more specialized immune markers because it is inexpensive, widely available, and repeatedly measured in routine care, although it remains a crude peripheral measure and does not directly characterize intratumoral immune function. Lymphopenia, commonly operationalized as an ALC below a threshold defined by study investigators, may be present at baseline or may emerge during therapy. Baseline lymphopenia can reflect heterogeneous host and disease characteristics, including prior systemic therapy or radiotherapy exposure, systemic inflammation, malnutrition, infection, bone marrow involvement, or use of lymphocyte-suppressing medications [8-10]. By contrast, on-treatment lymphopenia may also relate to the treatment course, intercurrent events, evolving disease status, or changes in supportive care. Because lymphocytes are central to immune-mediated tumor control and to the pharmacodynamic intent of checkpoint blockade, ALC-based lymphopenia has been evaluated as a routinely available marker that could capture interindividual variation in the host immune compartment in patients receiving ICIs [7,9].

A growing body of primary studies has evaluated lymphopenia in relation to clinical outcomes among adults with solid tumors treated with ICIs, mainly focusing on OS and PFS. However, this evidence spans heterogeneous tumor sites, therapeutic settings, and ICI classes, and the consistency of reported associations remains uncertain [11-13]. Related quantitative syntheses have attempted pooling in narrower contexts, particularly radiation-induced lymphopenia or radiotherapy/immunochemotherapy settings, but these address partly different exposure constructs and treatment contexts rather than ALC-defined lymphopenia as a prognostic exposure across ICI-treated solid tumors [14,15]. Methodological heterogeneity further limits comparability. Studies differ in how lymphopenia is defined, which ALC cut-offs are used, whether ALC is modeled as binary or continuous, and whether related immune indices are used instead. The timing of ALC assessment also varies, including baseline, early on-treatment, and dynamic follow-up evaluations, and baseline and on-treatment lymphopenia are not always analyzed as distinct exposures [16-18]. Analytical approaches also differ in eligibility criteria, follow-up, covariate adjustment, and outcome modeling. Together, these differences limit synthesis and create uncertainty about the prognostic relevance of lymphopenia during ICI therapy. This review addresses the gap by synthesizing the prognostic evidence on ALC-defined lymphopenia in solid tumors treated with ICIs while explicitly accounting for differences in timing, exposure definition, and analytical approach.

Given these uncertainties, a systematic review is warranted to consolidate the literature and to characterize heterogeneity in a transparent manner. A structured qualitative synthesis can document how lymphopenia has been defined and timed across studies, distinguish evidence pertaining to baseline versus on-treatment lymphopenia, and map tumor and treatment contexts in which ALC-based lymphopenia has been evaluated under ICI therapy. Clinically, clarifying this evidence may help determine whether ALC is best used as a descriptive prognostic covariate, a stratification variable in observational or trial analyses, or a longitudinal monitoring marker, while avoiding premature use for treatment selection. Consistent with a prognostic (non-causal) perspective, the purpose of this review is to evaluate associations rather than to infer causal effects, to define clinical decision thresholds, or to position lymphopenia as a predictive biomarker for treatment selection.

This systematic review evaluates whether baseline and/or on-treatment lymphopenia, defined in each study by an ALC below an author-specified threshold, is associated with survival outcomes in adults with solid tumors treated with ICIs. The primary outcome was OS, and the secondary outcome was PFS. Secondary objectives were to examine how reported associations varied according to timing of assessment, lymphopenia definitions and thresholds, tumor type, treatment context, and analytical approach.

## Review

Methods

Design, Reporting Framework, and Protocol

This systematic review was designed to evaluate the prognostic association between lymphopenia measured by peripheral ALC, assessed at baseline and/or during ICI therapy, and survival outcomes in adults with solid tumors. The review was conducted and reported in accordance with the Preferred Reporting Items for Systematic Reviews and Meta-Analyses (PRISMA) 2020 statement, using a prognostic (non-interventional) framework in which associations were summarized through a structured qualitative synthesis without causal inference. A protocol and analysis plan were developed a priori to define the eligibility criteria, outcomes, search approach, data extraction domains, risk-of-bias approach, and synthesis methods; the analysis framework is summarized in Supplementary Material 4. No eligibility criteria, outcome definitions, or broad synthesis plans were changed after title and abstract screening began. The data extraction form, risk-of-bias tool assignment rules, and study categorization framework were finalized before full-text assessment and extraction. The protocol was not registered in PROSPERO because the review was initiated as an exploratory synthesis before prospective registration was considered; this is acknowledged as a limitation that may reduce transparency regarding deviations from the original analysis plan.

Eligibility Criteria

Studies were eligible if they included adults (≥18 years) with solid tumors treated with ICIs targeting PD-1, PD-L1, and/or CTLA-4, administered as monotherapy or in combination. The eligible clinical setting was locally advanced or metastatic disease. Disease stage was ascertained from the original study reports; when staging was not explicitly reported or populations were clinically mixed, this was noted and considered during synthesis. The primary exposure of interest was lymphopenia, defined by an author-specified peripheral ALC threshold, assessed prior to ICI initiation (baseline) and/or during ICI therapy (on-treatment or dynamic lymphopenia). The primary comparator was the absence of lymphopenia at the corresponding timepoint. Studies evaluating related but non-equivalent immune constructs, such as derived leukocyte ratios or multivariable composite scores, were considered only for contextual qualitative description when they informed interpretation of the ALC literature; they were not considered equivalent to ALC-defined lymphopenia for the primary synthesis.

The primary outcome was OS. PFS was a key secondary time-to-event outcome. Time-to-progression (TTP), when reported as the primary progression-related endpoint in the absence of a PFS definition, was accepted as a related but non-equivalent measure and was described separately in the synthesis rather than treated as interchangeable with PFS. Additional outcomes of interest, when reported, were objective response rate (ORR), disease control rate (DCR), time-to-treatment failure (TTF), time-to-treatment discontinuation (TTD), time to next treatment (TTNT), or closely related endpoints, as well as discontinuation of ICI due to progression or clinical deterioration. Outcomes primarily related to toxicity were not within the scope of this review.

Eligible study designs comprised retrospective or prospective cohort studies, registry-based analyses, and subgroup or post hoc analyses of clinical trials, provided that the report evaluated the association between ALC-defined lymphopenia and oncologic outcomes under ICI therapy. Reports were restricted to full-text articles in English. Studies were excluded from the primary synthesis if they were narrative reviews, editorials, commentaries or letters, preclinical or animal studies, pediatric studies, isolated case reports/case series with fewer than 10 patients, studies focusing exclusively on lymphopenia induced by radiotherapy or chemotherapy without ICI exposure, studies not reporting any oncologic clinical outcomes, or studies evaluating only derived immune indices without a directly reported ALC-defined lymphopenia exposure. A low minimum cohort threshold was retained to avoid excluding sparse but potentially informative prognostic literature a priori; however, small studies were interpreted cautiously through the risk-of-bias assessment and lower-certainty evidence classification.

For synthesis planning, studies were first categorized by the timing of lymphopenia assessment (baseline versus on-treatment) and then by exposure construct (direct ALC-defined lymphopenia versus related derived immune constructs). Baseline lymphopenia was prespecified as ALC measured before ICI initiation. In contrast, on-treatment or dynamic lymphopenia was prespecified as ALC measured after treatment initiation, including early landmark or follow-up assessments as defined in the source studies. Exact timing windows were extracted as reported rather than forced into a single harmonized window, because the primary studies used heterogeneous landmark definitions. Baseline and on-treatment lymphopenia were treated as distinct exposures and were synthesized separately. Direct ALC-defined lymphopenia studies formed the primary evidence base of the review, whereas studies based on derived immune constructs were handled in a separate contextual category to avoid conflating non-equivalent prognostic measures.

Information Sources and Search Strategy

The search was conducted in PubMed (via NCBI), EMBASE, Scopus (via Elsevier), and Web of Science (via Clarivate). The most recent search of each database was performed on 5 January 2026, with no start-date restriction applied. The search strategy combined terms for ICIs and immunotherapy (including PD-1, PD-L1, and CTLA-4) with terms for lymphopenia and ALC, implemented using database-appropriate syntax. Free-text strings used to operationalize these concepts included (“immune checkpoint inhibitor” OR immunotherapy OR PD-1 OR PD-L1 OR CTLA-4) AND (lymphopenia OR lymphopaenia OR “absolute lymphocyte count” OR ALC), (“immune checkpoint inhibitor” OR immunotherapy) AND (lymphopenia OR “absolute lymphocyte count”) AND (survival OR prognosis OR outcome), immunotherapy AND (baseline lymphopenia OR treatment-induced lymphopenia OR early lymphopenia), and “absolute lymphocyte count” AND immunotherapy AND survival. Targeted searches incorporating tumor terms (e.g., melanoma, lung cancer) were also used to support retrieval where appropriate. Search results and cited references were reviewed for recent systematic reviews, meta-analyses, pooled analyses, and large retrospective or prospective cohorts relevant to baseline or on-treatment lymphopenia in ICI-treated solid tumors. Reports focused primarily on radiotherapy-induced lymphopenia, treatment toxicity without prognostic ICI-specific ALC analyses, hematologic malignancies, or non-ICI treatment contexts were considered contextual rather than eligible for the primary synthesis. Reproducible electronic search strategies for each database are provided in Supplementary Material 1, and the main reproducibility elements are summarized in Supplementary Material 11.

Selection Process

After de-duplication, two reviewers (I.B. and B.C.) independently screened titles and abstracts against the eligibility criteria. Full-text articles were retrieved for any record deemed potentially eligible by either reviewer and were assessed independently by the same two reviewers. Disagreements at the title and abstract or full-text stage were discussed between the two reviewers until consensus was reached; when consensus could not be reached, a third reviewer (H.M.) arbitrated the final eligibility decision. The reason for exclusion was recorded at the full-text stage. No automation tools were used for screening.

Data Collection Process and Data Items

Two reviewers (I.B. and B.C.) independently extracted data using a standardized form, with discrepancies resolved by consensus or adjudication by H.M. Data extraction disagreements were checked against the source article, discussed by the two reviewers, and referred to H.M. when consensus was not reached. When multiple publications reported overlapping cohorts, a single report per cohort was retained, prioritizing the most recent publication or the report with the largest sample size and most complete outcome reporting. When a study reported multiple effect estimates for the same outcome, one estimate was selected using prespecified rules that prioritized the most fully adjusted model and the exposure definition most closely aligned with a direct binary ALC-defined lymphopenia contrast.

Extracted items included study and setting characteristics (design, setting, recruitment period), tumor type and disease context, ICI regimen characteristics (agent class and regimen), lymphopenia definition (ALC threshold and units) and measurement timing, exposure construct (direct ALC-defined lymphopenia versus related derived construct), and outcome definitions. For OS and PFS, hazard ratios (HRs) and 95% confidence intervals (CIs) were extracted, prioritizing multivariable-adjusted HRs when available. Univariable HRs, median survival times, and log-rank statistics were retained for descriptive reporting when necessary but were interpreted separately from adjusted effect estimates. Effect estimates were not reconstructed from Kaplan-Meier curves or derived from scanned figures. For outcomes reported as proportions, such as ORR and DCR, effect measures were extracted as reported.

Risk-of-Bias Assessment

Risk of bias was assessed using tools selected according to study type and analytical framework. For non-randomized cohort studies evaluating lymphopenia as a binary prognostic exposure, the Risk of Bias In Non-randomized Studies of Interventions (ROBINS-I) was adapted from its original interventional framework to provide a structured domain-based assessment [19]. The assessed ROBINS-I domains were confounding, selection of participants, classification of exposures, missing data, measurement of outcomes, and selection of the reported result; the domain “departures from intended exposures” was considered inapplicable in this prognostic context and was not assessed. This adaptation beyond the original interventional scope of ROBINS-I is acknowledged as a methodological limitation. ROBINS-E was not adopted because the review was framed primarily as a prognostic synthesis rather than an assessment of causal exposure effects, and the Quality In Prognosis Studies (QUIPS) tool remained the most appropriate reference for prognostic factor studies [20]. QUIPS was used for prognostic factor studies in which the analysis was primarily framed as evaluation of the independent contribution of ALC to outcome prediction or when an explicit exposed-versus-reference comparison was not the dominant analytical structure. QUIPS was not applied to all studies because several reports were structured as exposed-versus-reference comparisons of lymphopenia groups rather than broad prognostic factor studies, whereas three reports primarily developed or evaluated prediction models and were better aligned with the Prediction model Risk of Bias Assessment Tool (PROBAST). The PROBAST was used for studies that primarily reported multivariable prognostic models [21]. Assignment of ROBINS-I versus QUIPS was based on the predominant analytical framing of each report: ROBINS-I was applied when outcomes were explicitly compared between a lymphopenia-defined group and a reference group, whereas QUIPS was applied when the prognostic factor framework was judged more appropriate. The decision algorithm used for tool assignment is provided in Supplementary Material 5. Although this rule improved consistency, some borderline studies remained difficult to classify, and this may have introduced limited heterogeneity in risk-of-bias assessment.

Two reviewers (I.B. and B.C.) independently assessed the risk of bias for each included study using the applicable tool; disagreements were resolved by consensus after reviewing the relevant tool domains, with arbitration by H.M. when consensus was not reached. Formal inter-rater agreement statistics, including kappa values, were not calculated because risk-of-bias judgments were finalized through consensus across different tools rather than analyzed as a single categorical rating exercise; this is acknowledged as a limitation of the review process. Where overall judgments were generated, they followed the decision rules of the respective tool. Prognostic model studies assessed with PROBAST and studies judged at high risk of bias were retained for qualitative interpretation but were considered lower-certainty components of the evidence base. Domain-level risk-of-bias judgments are summarized in Supplementary Material 6.

Effect Measures and Synthesis Methods

For OS and PFS, the principal effect measure extracted from the included studies was the HR comparing patients with lymphopenia versus those without lymphopenia, or the closest reported contrast when studies used related categorizations of ALC. When studies reported contrasts in the direction of higher ALC versus lower ALC rather than lymphopenia versus no lymphopenia, the original coding direction was retained and reported as published; HRs were not arithmetically inverted to a common reference direction. Adjusted HRs with 95% CIs were prioritized for interpretation, and unadjusted effect estimates were described separately when adjusted estimates were unavailable. P-values were recorded when reported by source studies, but they were not used as the primary basis for synthesis because their interpretation varied with model specification, sample size, and adjustment strategy. ORR, DCR, TTF, and other non-time-to-event outcomes were extracted as reported and summarized descriptively.

All included studies contributed to a structured qualitative synthesis. The synthesis was organized according to the timing of lymphopenia assessment (baseline versus on-treatment), tumor type and treatment context, ALC threshold and exposure definition, analytical approach, and risk-of-bias profile. Baseline and on-treatment lymphopenia were treated as distinct prognostic exposures throughout the review. Studies evaluating related derived immune constructs were summarized in a separate contextual category and were not interpreted as directly equivalent to ALC-defined lymphopenia.

No quantitative pooling was undertaken because of marked clinical and methodological heterogeneity across studies, including differences in tumor populations, ICI regimens, ALC thresholds, timing of assessment, exposure modeling, covariate adjustment, and outcome definitions. Exploratory meta-analysis was considered for baseline lymphopenia and OS, baseline lymphopenia and PFS, on-treatment lymphopenia and survival outcomes, and subgroups with apparently similar thresholds, such as ALC <1000 cells/µL or selected tumor types. However, these candidate groupings remained too sparse and non-commensurable for a defensible pooled estimate because studies differed in tumor setting, ICI regimen, baseline versus early-treatment exposure timing, threshold definition, coding direction, adjusted versus unadjusted estimates, and whether progression outcomes were reported as PFS or TTP. Random-effects models, Hartung-Knapp adjustment, meta-regression by threshold or timing, and threshold-stratified sensitivity analyses were therefore judged to be underpowered and at high risk of yielding misleading precision estimates. Because no pooled meta-analysis was performed, heterogeneity statistics such as I² and Cochran’s Q, model comparisons between fixed-effect and random-effects approaches, and funnel plot or regression-based publication-bias tests were not calculated. Vote-counting based solely on the direction of effect was not used as a formal synthesis method because it ignores study size, uncertainty, risk of bias, and effect magnitude; however, directionality was summarized narratively. The review, therefore, emphasizes patterns of consistency, divergence, and contextual variation rather than a single summary estimate. The feasibility assessment for candidate quantitative syntheses is summarized in Supplementary Material 12. Before final data extraction and synthesis, each study was assigned to one of four prespecified interpretive categories: “Primary ALC evidence” for direct ALC-defined lymphopenia contrasts using a binary threshold with at least one extractable survival estimate; “Lower-certainty ALC evidence” for direct but methodologically limited ALC-based contrasts, including very small cohorts, exclusively unadjusted analyses, incomplete effect reporting, or exploratory ALC categorizations; “Contextual ALC evidence” for ALC-based categorizations that did not use a fixed binary lymphopenia threshold; and “Derived contextual evidence” for immune constructs derived from, but not equivalent to, direct ALC. The first two categories formed the main qualitative evidence base, whereas the latter two were retained for contextual interpretation only. Contextual and derived contextual studies were evaluated for directional consistency with the primary ALC-defined evidence base and for identification of additional sources of heterogeneity, but they were not incorporated into summary statements about the prognostic relevance of ALC-defined lymphopenia. Where relevant, the direction and magnitude of reported associations were described narratively, with attention to adjustment status, exposure definition, and timing of assessment.

Because several studies reported inverse contrasts, such as higher ALC versus lower ALC rather than lymphopenia versus no lymphopenia, published HRs were not arithmetically inverted in the main synthesis. Instead, the contrast direction was preserved as reported, and an additional harmonized directional summary was prepared to indicate whether the published findings were directionally adverse for lower ALC or for lymphopenia. This harmonized interpretation is provided in Supplementary Material 9 and should be read as a qualitative directional aid rather than as a recalculated effect-estimate table.

Reporting Bias and Certainty Assessment

Because no quantitative pooling was undertaken for the primary synthesis, formal statistical assessment of small-study effects was not performed. No formal certainty-of-evidence framework, such as GRADE adapted for prognostic factor reviews, was prespecified or applied. A formal GRADE adaptation was not attempted because the evidence was not organized around a single pooled effect estimate and because exposure definitions, timing, thresholds, analytical contrasts, and adjustment strategies varied substantially across studies. Confidence in the body of evidence was therefore appraised qualitatively, with attention to the risk of bias, consistency of findings, directness of the exposure definition, precision and reporting completeness, and clinical and methodological heterogeneity. Narrative certainty judgments are summarized in Supplementary Material 8. This approach is less standardized than a formal GRADE assessment and is acknowledged as a limitation of the review.

Results

Study Selection

**Figure 1:**
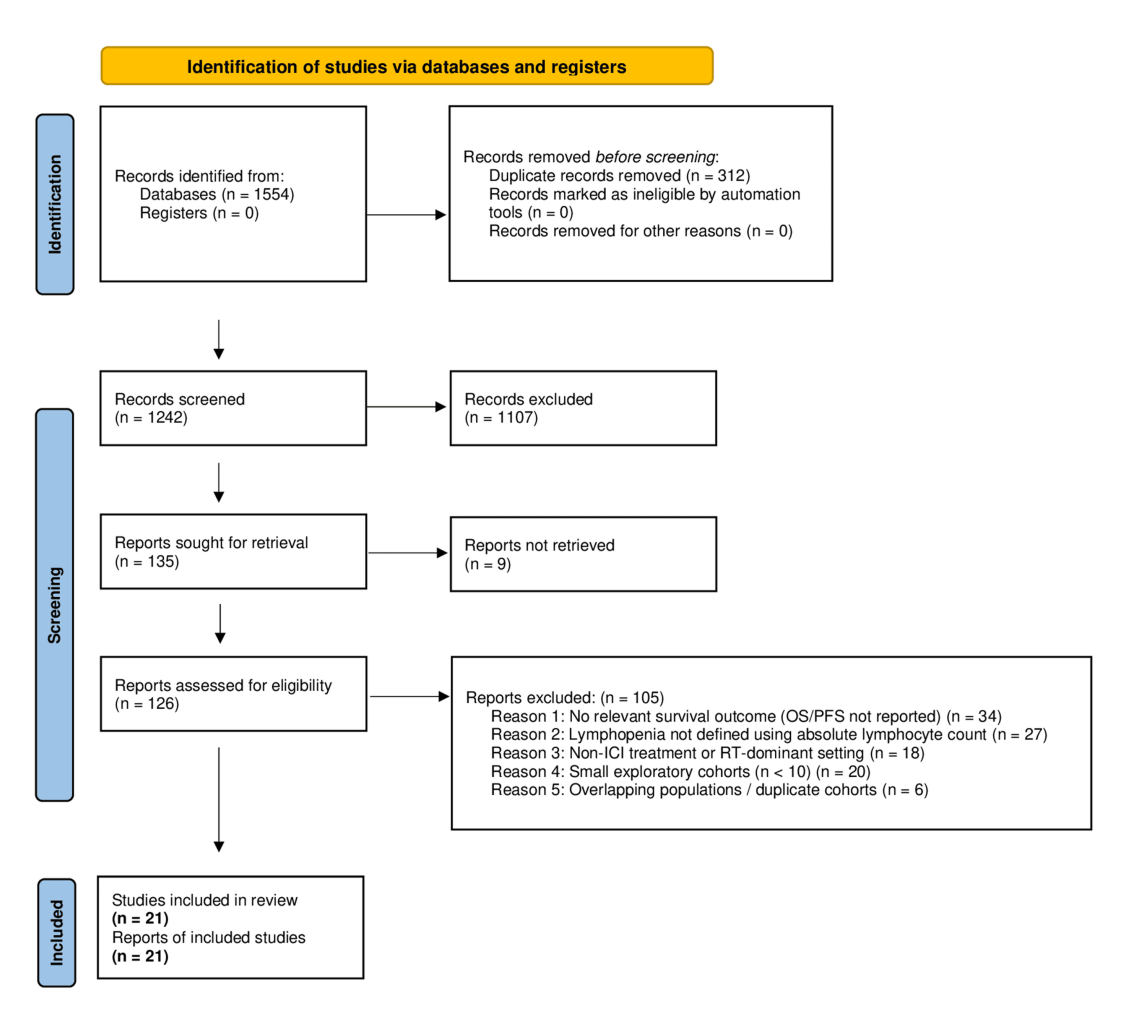
PRISMA 2020 flow diagram of study selection. PRISMA: Preferred Reporting Items for Systematic Reviews and Meta-Analyses

The search identified 1,554 records from bibliographic databases and no records from registers (Figure 1). After removal of 312 duplicate records (and with no records excluded by automation tools or other reasons prior to screening), 1,242 records were screened at the title and abstract level, and 1,107 were excluded. Full texts were sought for 135 reports; nine reports could not be retrieved. A total of 126 reports were assessed for eligibility at the full-text level. Of these, 105 reports were excluded, most commonly because relevant survival outcomes were not reported (OS and/or PFS; n=34), lymphopenia was not defined using ALC (n=27), the treatment setting was non-ICI or radiotherapy-dominant (n=18), cohort size was <10 (n=20), or overlapping populations/duplicate cohorts were identified (n=6). Twenty-one studies (21 reports) met the eligibility criteria and were included in the review. Fifteen studies reporting direct ALC-defined lymphopenia contrasts contributed to the primary synthesis (12 classified as primary ALC evidence and three as lower-certainty ALC evidence), whereas six studies based on ALC categorizations without a fixed binary threshold or on derived immune constructs were retained for contextual interpretation only.

Characteristics of the Included Studies

The 21 studies encompassed a broad range of solid malignancies and immune checkpoint inhibitor regimens, including non-small cell lung cancer [22], melanoma [23], renal cell carcinoma [24], esophageal squamous cell carcinoma [25], head and neck squamous cell carcinoma [18], and mixed solid tumor cohorts [16]. Sample sizes ranged from 22 patients in a small retrospective cohort [26] to 18,186 patients in a registry-based observational analysis [27]. Across studies, immune checkpoint inhibitor regimens included PD-1 inhibitors (nivolumab, pembrolizumab), PD-L1 inhibitors (atezolizumab), CTLA-4 inhibitors (ipilimumab), and combination regimens. The identical sample size reported in Sun et al. (2017) and Diehl et al. (2017) (n = 167) was noted during extraction; review of the published study characteristics indicated distinct institutional settings and recruitment windows, and no evidence of cohort overlap was identified [16,28].

Lymphopenia was defined using author-specified ALC thresholds. Common cut-offs were 1,000 cells/µL or 1.0 × 10⁹/L [12], with some studies applying lower thresholds (e.g., 500 cells/µL) [29] and others using quantile-based categorizations [30]. The timing of ALC assessment included baseline (pre-treatment) and early on-treatment timepoints (e.g., 3 or 6 weeks). Median follow-up ranged from 6.4 months [11] to over 23 months among studies reporting follow-up [31]. These characteristics are summarized in Table 1, which prioritizes direct ALC-defined lymphopenia studies while retaining related derived immune constructs for contextual interpretation. A dedicated summary of thresholds and timing windows is provided in Supplementary Material 10.

**Table 1:** Characteristics of the included studies. Direct ALC-defined lymphopenia studies form the primary evidence base of this review; studies based on derived immune constructs or dynamic model-based measures were retained for contextual interpretation. Units and thresholds are reported as described in the source studies. ALC: absolute lymphocyte count; OS: overall survival; PFS: progression-free survival; ORR: objective response rate; TTNT: time to next treatment; TTP: time-to-progression; TTD: time-to-treatment discontinuation; DCR: disease control rate.

Study (Author, Year)	Study Type	Cancer Type	n	Exposure Construct	Definition/Timing	Key Outcomes	Main Qualitative Signal	Role in Review
Cho et al. (2019) [11]	Retrospective cohort	Advanced non-small cell lung cancer (NSCLC)	268	Direct ALC-defined lymphopenia	ALC <1000 cells/mm³; baseline/early treatment	OS, PFS	Lower ALC associated with worse OS and PFS	Primary ALC evidence
Takemura et al. (2024) [12]	Registry-based cohort	Metastatic renal cell carcinoma (RCC)	966	Direct ALC-defined lymphopenia	Baseline ALC <1000 cells/µL	OS, ORR, TTNT	Baseline lymphopenia associated with worse OS	Primary ALC evidence
Diehl et al. (2017) [16]	Retrospective cohort	Mixed solid tumors	167	Direct ALC-defined lymphopenia	ALC <1000 cells/µL; baseline and 3 months	TTP	Lower ALC associated with worse baseline and 3-month time-to-progression	Primary ALC evidence
Postow et al. (2020) [17]	Retrospective RCT analysis	Advanced melanoma	1136	Direct ALC-defined lymphopenia	Baseline and week 6 ALC at 1.0 × 10⁹/L	OS	Higher ALC associated with better OS in trial-derived data; reported inversely	Primary ALC evidence
Park et al. (2020) [18]	Retrospective cohort	HNSCC	108	Direct ALC-defined lymphopenia	Week 6 low ALC <770 cells/µL	PFS, OS, ORR	Week 6 ALC associated with PFS; adjusted HR not directly extractable	Primary ALC evidence
Karantanos et al. (2019) [26]	Retrospective cohort	Advanced NSCLC	22	Exploratory ALC categorization	Baseline quartiles and week 6 ALC	OS, time on treatment	Exploratory signal only; very small unadjusted study	Lower-certainty ALC evidence
Lee et al. (2022) [30]	Retrospective cohort	NSCLC	231	Post-treatment ALC category	Post-treatment quartiles at 3 to 4 weeks	PFS, OS, ORR	Highest post-treatment quartile associated with better PFS and OS	Contextual ALC evidence
Goldschmidt et al. (2023) [27]	Electronic health record (EHR)-based retrospective cohort	Melanoma, NSCLC, RCC	18186	ALC quintile categorization	Lowest vs highest ALC quintiles by tumor type	OS, TTD, TTNT	Lowest ALC categories associated with worse OS across several tumor types	Contextual ALC evidence
Zhen et al. (2024) [22]	Prognostic model study	NSCLC	81	Dynamic ratio construct	Ratio of post-cycle to baseline counts	Response	Dynamic response-oriented ratios reported; not directly comparable with binary ALC	Derived contextual evidence
Foerster et al. (2025) [32]	Retrospective cohort	Stage IV melanoma	141	Direct ALC-defined lymphopenia	Baseline ALC dichotomized at 1.61 × 10³/µL	PFS, OS, ORR	Higher ALC associated with better PFS; inverse coding requires careful interpretation	Primary ALC evidence
Tomsitz et al. (2022) [23]	Retrospective cohort	Metastatic melanoma	116	On-treatment ALC-defined lymphopenia	Lymphopenia <1000 cells/µL at any time	PFS, OS	Developed lymphopenia associated with shorter unadjusted PFS and OS	Lower-certainty ALC evidence
Ueda et al. (2023) [24]	Prognostic model study	Advanced RCC	46	Direct pretreatment ALC category	Pretreatment low ALC <1289 cells/µL	PFS, OS, ORR	Lower pretreatment ALC associated with worse outcomes, but the model risk was high	Lower-certainty ALC evidence
Yin et al. (2022) [29]	Retrospective cohort	Esophageal squamous cell carcinoma (ESCC)	167	Direct ALC-defined lymphopenia	ALC <0.50 × 10⁹/L; baseline/on-treatment	PFS, ORR, DCR	Lower ALC associated with worse PFS	Primary ALC evidence
Zhao et al. (2022) [25]	Retrospective cohort	ESCC	105	Direct ALC-defined lymphopenia	Baseline ALC ≤625 cells/µL	OS	Lower baseline ALC associated with worse OS	Primary ALC evidence
Ho et al. (2018) [33]	Retrospective cohort	Recurrent/metastatic head and neck squamous cell carcinoma (HNSCC)	34	Direct ALC-defined lymphopenia	Pretreatment ALC <600 cells/µL	PFS, ORR, DCR	Lower pretreatment ALC associated with worse PFS	Primary ALC evidence
Pike et al. (2019) [34]	Retrospective cohort	Mixed solid tumors	110	Direct ALC-defined lymphopenia	Severe lymphopenia: ALC <500 cells/µL at start	OS	Severe baseline lymphopenia associated with worse OS	Primary ALC evidence
Sun et al. (2017) [28]	Retrospective cohort	Various solid tumors	167	Direct ALC-defined lymphopenia	Baseline ALC <1 G/L	OS, ORR, DCR	Near-null baseline OS association	Primary ALC evidence
Conroy et al. (2024) [35]	Retrospective cohort	Mixed solid tumors	179	Direct ALC-defined lymphopenia	ALC <1.0 × 10⁹/L; baseline and 3 months	OS, PFS	Adverse 3-month association reported; adjusted HR incompletely reported	Primary ALC evidence
Du et al. (2023) [31]	Prognostic model study	NSCLC	146	Dynamic lymphocyte-change construct	Decreased lymphocyte counts after 12 weeks	PFS, OS, ORR	Dynamic post-baseline measures associated with outcomes; directly non-comparable	Contextual ALC evidence
Soyano et al. (2018) [36]	Retrospective cohort	NSCLC	157	Derived leukocyte ratio	Baseline absolute neutrophil count (ANC):ALC ratio ≥5.9	OS, PFS, immune-related adverse events	Ratio-based construct associated with worse OS	Derived contextual evidence
Maymani et al. (2018) [37]	Retrospective subgroup analysis	NSCLC	74	Derived leukocyte ratio	Baseline neutrophil-to-lymphocyte ratio (NLR) >6	OS, PFS	Ratio-based construct associated with worse OS in univariate analysis	Derived contextual evidence

Risk of Bias in the Included Studies

Based on the risk-of-bias tool assignments provided for this review, 12 studies were assessed with ROBINS-I, six with QUIPS, and three with PROBAST. Overall study-level risk of bias was judged as low for one study [17], moderate for 16 studies, serious for one study [26], and high for three studies [22]. These overall judgments are tool-specific and should not be interpreted as directly comparable across ROBINS-I, QUIPS, and PROBAST. Across the evidence base, 10 studies contributed at least one adjusted survival estimate, five contributed only unadjusted or incompletely adjusted time-to-event information, and six contributed contextual or otherwise non-directly comparable evidence. Moderate-risk judgments were mainly related to retrospective design features and incomplete control of confounding. The serious-risk judgment for Karantanos et al. (2019) [26] was attributed to a small sample size and reliance on univariate analyses without adjustment for key prognostic factors. High-risk judgments for the three prognostic model studies were attributed to low events-per-variable ratios and a lack of adjustment for overfitting. These study-level risk-of-bias assessments are summarized in Table 2.

**Table 2:** Risk-of-bias assessment of the included studies. For the purposes of this qualitative synthesis, low- and moderate-risk studies were considered more directly informative, whereas serious or high-risk studies were interpreted more cautiously. ROBINS-I: Risk of Bias in Non-randomized Studies of Interventions; QUIPS: Quality in Prognosis Studies; PROBAST: Prediction Model Risk of Bias Assessment Tool.

Study (Author, Year)	Study Type	Tool	Main Limitation for Interpretation	Overall Risk of Bias
Cho et al. (2019) [11]	Observational retrospective cohort	ROBINS-I	Residual confounding despite multivariable adjustment	Moderate
Conroy et al. (2024) [35]	Observational retrospective cohort	ROBINS-I	Incomplete reporting of adjusted contrasts	Moderate
Diehl et al. (2017) [16]	Observational retrospective cohort	ROBINS-I	Post-baseline analyses may be vulnerable to time-related bias	Moderate
Foerster et al. (2025) [32]	Observational retrospective cohort	ROBINS-I	Exploratory cut-off selection	Moderate
Ho et al. (2018) [33]	Observational retrospective cohort	ROBINS-I	Small sample and residual confounding	Moderate
Karantanos et al. (2019) [26]	Observational retrospective cohort	ROBINS-I	Very small sample and univariate analyses without adequate adjustment	Serious
Pike et al. (2019) [34]	Observational retrospective cohort	ROBINS-I	Threshold-dependent exposure definition in a retrospective setting	Moderate
Postow et al. (2020) [17]	Retrospective analysis of randomized controlled trial (RCT) data	ROBINS-I	Lowest risk in the review; contrast direction still requires careful reading	Low
Sun et al. (2017) [28]	Observational retrospective cohort	ROBINS-I	Retrospective phase I population	Moderate
Yin et al. (2022) [29]	Observational retrospective cohort	ROBINS-I	Retrospective design and treatment heterogeneity	Moderate
Park et al. (2020) [18]	Prognostic retrospective cohort	QUIPS	Moderate confounding concern in post-baseline analysis	Moderate
Du et al. (2023) [31]	Prognostic model development	PROBAST	Model-development analysis with high risk of overfitting	High
Lee et al. (2022) [30]	Prognostic retrospective cohort	QUIPS	Post-treatment quartile exposure and retrospective confounding	Moderate
Soyano et al. (2018) [36]	Prognostic retrospective cohort	QUIPS	Derived ratio exposure rather than direct ALC-defined lymphopenia	Moderate
Maymani et al. (2018) [37]	Prognostic retrospective subgroup study	QUIPS	Small sample and derived ratio exposure	Moderate
Zhao et al. (2022) [25]	Observational retrospective cohort	ROBINS-I	Residual confounding in a restricted disease setting	Moderate
Goldschmidt et al. (2023) [27]	EHR-based retrospective cohort	QUIPS	Missing EHR covariates despite a large sample	Moderate
Takemura et al. (2024) [12]	Observational registry-based cohort	ROBINS-I	Residual confounding in a registry-based exposed-versus-reference comparison	Moderate
Tomsitz et al. (2022) [23]	Prognostic retrospective cohort	QUIPS	Adjusted estimates not directly reported for ALC-defined contrasts	Moderate
Ueda et al. (2023) [24]	Prognostic model development	PROBAST	Very small derivation cohort with high overfitting risk	High
Zhen et al. (2024) [22]	Prognostic prediction model	PROBAST	Small model-development cohort and risk of overfitting	High

Baseline Lymphopenia and OS

Reported associations between baseline ALC-defined lymphopenia and OS were unfavorable in several cohorts, but not fully consistent across tumor types, thresholds, and analytical strategies. Among studies reporting adjusted baseline estimates using direct ALC-defined contrasts, Cho et al. (2019) [11], Pike et al. (2019) [34], Takemura et al. (2024) [12], and Zhao et al. (2022) [25] each reported worse OS in patients with lymphopenia; where directly extractable, adjusted estimates were directionally adverse and above 1.0. Goldschmidt et al. (2023) [27] likewise reported less favorable OS in the lowest ALC categories across several tumor types in an EHR-based cohort.

However, not all baseline findings were aligned in the same format or direction of reporting. Sun et al. (2017) [28] reported a near-null association, whereas Postow et al. (2020) [17] reported an inverse contrast based on ALC greater than or equal to 1.0 × 10⁹/L versus lower ALC in trial-derived melanoma data. These reporting differences, together with variation in threshold selection, adjustment strategy, and study population, limited direct comparability of baseline OS findings across studies. Directionality of reported contrasts is summarized in Supplementary Material 2.

A harmonized qualitative direction table is also provided in Supplementary Material 9 to help readers interpret whether each reported contrast was directionally adverse for lower ALC or lymphopenia. This table does not recalculate or invert HRs; it preserves published contrast directions while providing a common interpretive orientation.

Baseline Lymphopenia and PFS

PFS findings for baseline or early-treatment ALC-defined lymphopenia also varied by setting and by exposure definition. Cho et al. (2019) [11] reported an adjusted HR of 2.35 (95% CI 1.47-3.75) for lymphopenia versus no lymphopenia, and Ho et al. (2018) [33] reported a PFS HR of 3.65 (95% CI 1.05-12.98) in recurrent or metastatic HNSCC. Diehl et al. (2017) [16] reported an association between lower baseline ALC and worse TTP, while Ueda et al. (2023) [24] reported worse PFS in RCC patients with lower pretreatment ALC.

By contrast, some studies reported results using contrasts framed as higher versus lower ALC rather than lymphopenia versus no lymphopenia. Foerster et al. (2025) [32] reported a PFS HR of 0.549 (95% CI 0.361-0.833) for higher baseline ALC in stage IV melanoma, and Lee et al. (2022) [30] reported an adjusted PFS HR of 0.28 (95% CI 0.16-0.52) when comparing the highest versus the lowest post-treatment lymphocyte quartile in NSCLC. Diehl et al. (2017) contributed related progression evidence through TTP rather than formally defined PFS and was therefore interpreted as supportive but not directly interchangeable progression-related evidence. Taken together, these studies broadly suggest that lower lymphocyte counts tend to track with less favorable progression-related outcomes in several settings, while underscoring that exposure coding, outcome definition, and timing differed substantially across reports.

On-Treatment Lymphopenia and Survival Outcomes

Evidence on lymphopenia arising during treatment was more heterogeneous and was particularly sensitive to timing and analytical approach. Conroy et al. (2024) [35] described adverse OS associations for three-month lymphopenia in a mixed solid tumor cohort, although adjusted HRs were not consistently reported for all contrasts. Diehl et al. (2017) [16] found stronger associations at three months than at baseline for TTP, and Park et al. (2020) [18] reported significant adjusted associations between week 6 ALC and PFS in HNSCC.

Other reports assessed on-treatment lymphopenia using less directly comparable constructs. Tomsitz et al. (2022) [23] described shorter unadjusted PFS and OS among patients who developed lymphopenia during therapy, whereas Karantanos et al. (2019) [26] and Du et al. (2023) [31] used exploratory dynamic or post-baseline lymphocyte measures that are difficult to align with fixed binary landmark definitions. In summary, reported associations between on-treatment lymphopenia and survival outcomes were variable across studies and were assessed using different analytical approaches, timepoints, and exposure constructs.

Studies Using Related Derived Immune Constructs

Some included reports evaluated immune constructs related to, but not equivalent to, ALC-defined lymphopenia. Soyano et al. (2018) [36] and Maymani et al. (2018) [37] focused on leukocyte ratios rather than direct ALC-defined lymphopenia, and Zhen et al. (2024) [22] reported dynamic response-oriented ratios after treatment initiation. These studies were retained for contextual interpretation because they reflect the broader prognostic biomarker landscape in immuno-oncology, but they were not interpreted as direct evidence for the primary ALC-defined exposure.

As a sensitivity narrative, contextual ALC studies and derived immune-construct studies were compared directionally with the primary ALC-defined evidence base. Large ALC-categorization studies, particularly Goldschmidt et al. (2023) [27], were directionally consistent with the main qualitative pattern that lower lymphocyte counts tended to accompany less favorable outcomes. Post-treatment quartile-based ALC analyses also generally supported the same direction of association [30], although their exposure definitions were not interchangeable with fixed binary lymphopenia thresholds. Derived ratio studies were more indirect because they combined lymphocyte count with other inflammatory markers, but they did not suggest a contradictory overall pattern. Therefore, including contextual studies in a sensitivity narrative did not materially alter the interpretation of the primary synthesis, although it reinforced the importance of distinguishing direct ALC-defined lymphopenia from broader immune or inflammatory constructs.

Secondary Outcomes and Overall Synthesis Pattern

**Figure 2:**
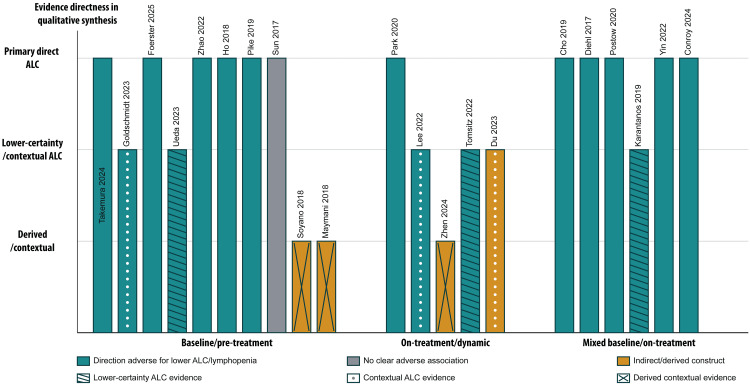
Unpooled harvest plot of ALC-related prognostic evidence by timing of assessment. Bar height reflects the role and directness of each study in the structured qualitative synthesis, with taller bars representing direct primary ALC-defined evidence. Colors indicate whether the qualitative direction was adverse for lower ALC or lymphopenia, showed no clear adverse association, or reflected an indirect or derived immune construct. Patterning distinguishes lower-certainty, contextual ALC, and derived contextual evidence. No pooled estimate is shown.

Secondary outcomes such as ORR, DCR, TTF, and TTNT were reported inconsistently and were less standardized than OS or PFS. Ho et al. (2018) [33] and Ueda et al. (2023) [24] reported ORR by pretreatment lymphopenia status, and Takemura et al. (2024) [12] reported ORR of 28% in the lymphopenia group and 37% in the non-lymphopenia group. Several studies reported associations between lower ALC and less favorable survival outcomes, although findings were heterogeneous and context-dependent. The strength, direction, and interpretability of associations varied materially according to tumor type, timing of assessment, exposure coding, and analytical rigor. Figure 2 provides an unpooled harvest plot summarizing the qualitative direction and directness of the evidence by timing of ALC assessment. The plot does not display a pooled estimate and should be interpreted as a visual summary of the structured qualitative synthesis rather than as a quantitative meta-analysis.

Main Sources of Heterogeneity Across Studies

The main sources of heterogeneity were clinical and methodological. Clinically, studies differed by tumor type, line of therapy, ICI regimen, disease burden, and prior treatment exposure. Methodologically, they differed in ALC thresholds, timing of ALC measurement, binary versus categorical or continuous exposure modeling, outcome definitions, and covariate adjustment strategies. These features limited cross-study comparability and support the interpretation of the available literature as context-dependent rather than reducible to a single summary effect. Threshold groupings used across studies are summarized descriptively in Supplementary Material 3.

Reporting Biases

Because the primary synthesis was qualitative, formal statistical assessment of reporting bias was not performed. Instead, the potential influence of selective reporting and small-study effects was considered indirectly through study design, risk-of-bias assessment, and variation in the completeness of adjusted effect reporting. Negative or null studies may remain unpublished, especially when ALC was examined as an exploratory laboratory variable in retrospective cohorts. In addition, smaller studies sometimes reported stronger or less stable associations, but the number of studies within comparable tumor, threshold, timing, and outcome strata was too limited to evaluate small-study effects formally. Selective reporting within retrospective cohorts also remains plausible because ALC thresholds, timing windows, and adjusted models may have been chosen or reported selectively. These concerns support cautious qualitative interpretation rather than a pooled estimate or a strong clinical conclusion.

Discussion

Interpretation in the Context of Other Evidence

Overall evidence pattern: this review addressed whether lymphopenia, defined by a low peripheral ALC, measured at baseline and/or during ICI therapy, is associated with survival outcomes in adults with solid tumors. Across the included studies, ALC-based lymphopenia was examined as a pragmatic prognostic factor, and the dominant qualitative pattern was that lower lymphocyte counts often tracked with less favorable outcomes. However, this pattern was not sufficiently consistent in design, coding, timing, or clinical context to support a single generalizable interpretation across all settings. Overall, lymphopenia appears better understood as a context-sensitive marker than as a uniform prognostic classifier for ICI-treated solid tumors.

Baseline versus on-treatment lymphopenia: temporality is central to interpreting this literature. Baseline lymphopenia and lymphopenia arising during treatment represent related but non-equivalent exposures that likely capture different clinical states. Baseline ALC integrates host reserve and disease characteristics present before immunotherapy initiation, including prior anticancer therapies, comorbidities, concomitant medications, systemic inflammation, and tumor burden [2,8,9]. In this setting, lymphopenia may act primarily as a summary marker of overall prognosis and treatment tolerance rather than as a direct determinant of ICI benefit. By contrast, on-treatment lymphopenia may reflect evolving disease course, intercurrent events, or treatment-related changes during follow-up, and its prognostic meaning is therefore harder to disentangle from the clinical trajectory. In some reports, on-treatment associations appeared stronger in magnitude than baseline associations, but their interpretability is more constrained by susceptibility to reverse causation, informative dropout, and time-dependent confounding.

This distinction is clinically important because lymphopenia may partly represent frailty, systemic inflammation, malnutrition, high disease burden, prior treatment exposure, or reduced physiological reserve rather than immune competence alone. Consequently, associations between low ALC and adverse survival should not be interpreted as evidence that lymphopenia itself causally reduces ICI efficacy. In many settings, ALC may function as an accessible marker of broader host and disease context.

Prognostic rather than predictive interpretation: the current evidence also does not establish whether lymphopenia predicts differential benefit from ICIs compared with other systemic therapies. Most included studies evaluated outcomes among ICI-treated patients without a non-ICI comparator arm capable of separating prognostic from predictive value. Accordingly, ALC-defined lymphopenia should be interpreted as a candidate prognostic marker in ICI-treated populations, not as evidence of treatment-specific predictive benefit.

Tumor and treatment heterogeneity: heterogeneity across tumor types and treatment contexts plausibly contributes to divergent findings. The included evidence spans multiple malignancies and ICI regimens, delivered in different lines of therapy and in populations with different baseline risks. Differences in eligibility criteria, prior treatment exposure, and supportive care practices can influence both ALC and outcomes and may not be fully captured by study-level adjustment. Methodological diversity further amplifies this variability, including different ALC thresholds, binary versus multi-category exposure definitions, and different time windows for ALC assessment. Some reports framed exposure as low versus high ALC, whereas others reported the inverse contrast, showing how coding choices and analytic approach can alter the apparent direction of prognostic relationships and complicate interpretation.

Biological plausibility and measurement limitations: biological considerations offer a rationale for why lymphopenia might correlate with outcomes under ICI therapy while also underscoring why a prognostic association should not be interpreted as causal. Lymphocytes, particularly T-cell populations, are integral to anti-tumor immunity and to the intended pharmacodynamic effect of checkpoint blockade [3,7]. Lower circulating lymphocyte counts may be consistent with reduced immune effector reserve, impaired priming capacity, or broader immunosuppressive states that accompany advanced disease or prior treatment exposure [9,38]. However, peripheral ALC is a coarse measure that may not reflect intratumoral immune composition, functional lymphocyte subsets, or spatial immune dynamics within the tumor microenvironment. Circulating lymphocyte counts can also vary with transient redistribution, infections, or medication effects, and the same ALC value may correspond to different underlying immune states across tumor types and clinical settings.

**Figure 3:**
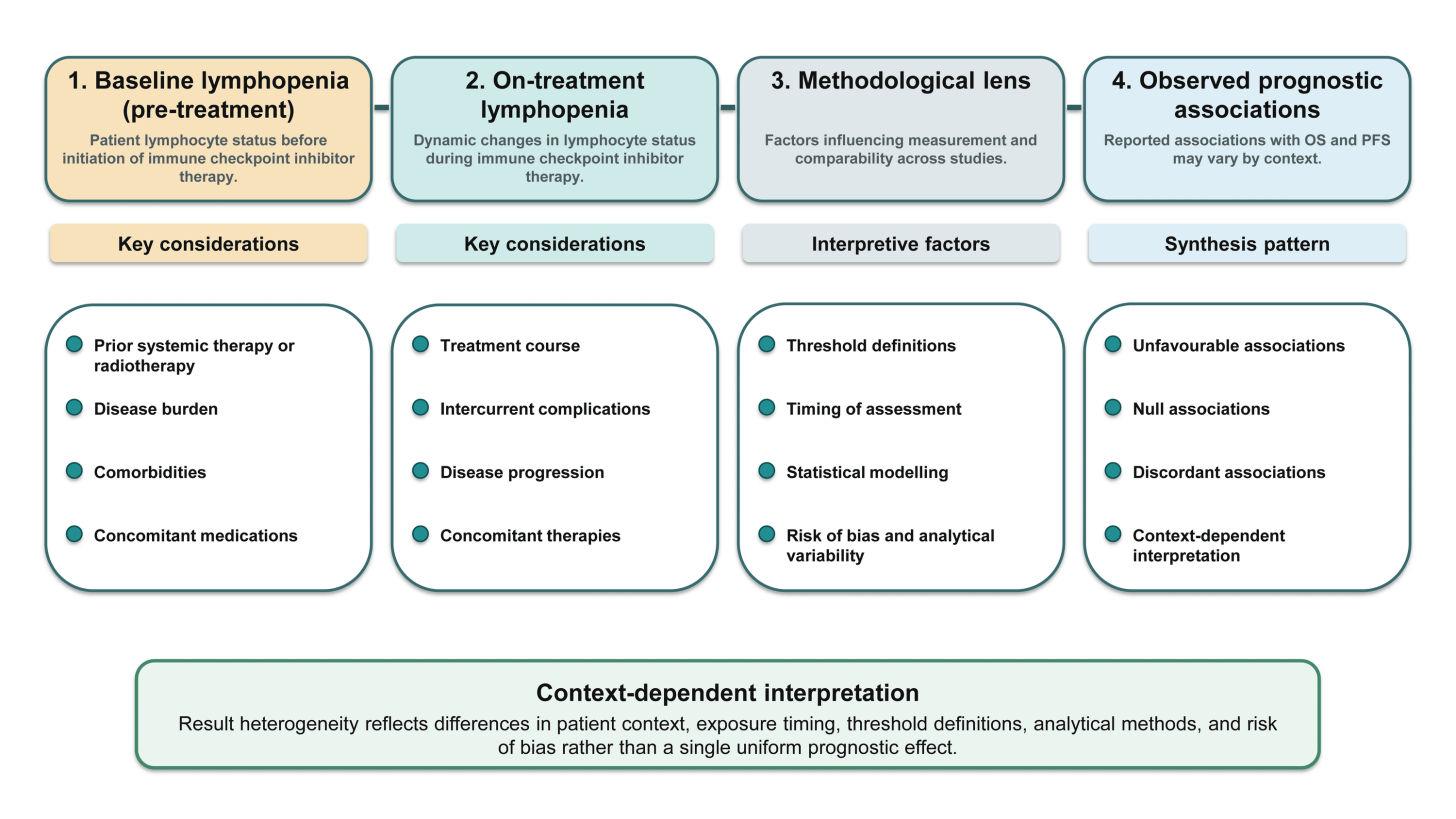
Conceptual framework linking baseline and on-treatment lymphopenia to prognostic heterogeneity during ICI therapy in solid tumors. The framework distinguishes baseline lymphopenia, reflecting host reserve and disease-related factors present prior to immunotherapy initiation, from lymphopenia occurring during treatment, which may capture dynamic clinical processes during follow-up. The methodological lens highlights how differences in lymphopenia definitions, timing of assessment, and statistical modeling influence comparability across studies. Observed associations with OS and PFS vary across contexts and may be unfavorable, null, or discordant, supporting the interpretation of lymphopenia as a context-dependent prognostic marker rather than a uniform prognostic classifier. The framework is intended to support interpretation of heterogeneous observational findings rather than to guide clinical decision-making. Figure created using Microsoft PowerPoint (Microsoft Corporation, Redmond, Washington). No AI-assisted image generation or editing was used.

Dynamic assessment and conceptual framework: interpretation of on-treatment lymphopenia is additionally constrained by time-dependent processes. When lymphopenia is assessed during therapy, it may be influenced by early progression, clinical deterioration, or changes in concomitant treatments, raising the possibility that observed associations partially reflect reverse causation [11,39]. Different analytic strategies, such as fixed landmark assessments versus time-varying approaches, address different questions and are not directly interchangeable. Figure 3 provides a conceptual framework that distinguishes baseline from on-treatment lymphopenia and highlights clinical and methodological factors potentially underlying heterogeneous prognostic associations.

Limitations of the Evidence

Confounding and study design: the evidence base has limitations that restrict the strength of conclusions. Most studies were observational, often retrospective, and therefore susceptible to confounding and selection mechanisms that are difficult to fully address with statistical adjustment. Baseline lymphopenia is likely correlated with performance status, comorbidity burden, tumor burden, prior systemic therapy and radiotherapy exposure, and concomitant immunomodulating medications; the extent and consistency of covariate capture and adjustment varied across studies. Even when multivariable estimates were reported, residual confounding remained plausible, particularly for factors that are incompletely measured in routine datasets. Covariate adjustment domains and residual confounding concerns are summarized in Supplementary Material 7.

Threshold and timing variability: substantial heterogeneity in exposure definition and timing further limits comparability. ALC thresholds differed across studies and were sometimes based on clinical grading cut-offs, cohort-specific distributions, or multi-level categorizations. Clinical grading systems, such as the Common Terminology Criteria for Adverse Events (CTCAE), could provide a standardized vocabulary for reporting lymphopenia severity. However, CTCAE grades are toxicity-oriented categories and may not fully address prognostic questions related to baseline immune reserve, longitudinal ALC change, landmark timing, and covariate adjustment. Dichotomization at different cut-offs can obscure dose-response relationships and may yield inconsistent findings when the underlying relationship is continuous or non-linear. This review did not perform threshold-stratified synthesis, despite the approximately three-fold range of ALC cut-offs used across studies, which may have obscured threshold-dependent prognostic gradients and limited identification of clinically meaningful boundaries. In addition, baseline and on-treatment lymphopenia were not consistently treated as distinct exposures, and the selection of early on-treatment timepoints differed, complicating any synthesis of dynamic effects. Outcome definitions and ascertainment practices, especially for PFS, can vary across settings and may be less robust in retrospective cohorts with non-standardized imaging schedules.

Risk of bias and reporting limitations: risk-of-bias assessments indicated recurrent concerns across domains relevant to prognostic research, including confounding, participant selection, and selective reporting of results. A subset of studies focusing on prognostic model development was judged to be at high risk of bias, reflecting vulnerabilities such as limited events relative to the number of predictors and the risk of overfitting. These features limit generalizability and constrain how model-based findings can be integrated with prognostic factor estimates from traditional cohort analyses. As a sensitivity consideration, the inclusion of Karantanos et al. (2019), which was judged to be at serious risk of bias due to its very small sample size and unadjusted analyses, did not materially influence the qualitative synthesis, as it was prespecified as lower-certainty exploratory evidence and was not incorporated into summary statements. Taken together, these limitations reinforce the need to interpret the current evidence base qualitatively, with attention to study design and directness of the reported ALC construct, rather than as a single unified body of interchangeable estimates.

Interpretation of reported HRs is also limited by incomplete reporting of model diagnostics in the primary studies. In particular, verification of the proportional hazards assumption was not consistently described, and violations of this assumption are plausible for on-treatment lymphopenia analyses in which prognostic effects may vary over time.

Limitations of the Review Processes

Limitations of the review processes may have affected completeness and interpretability. Although the work plan was developed a priori, the protocol was not registered in PROSPERO, which may be perceived as limiting transparency about prespecified decisions and deviations. The search was restricted to four bibliographic databases and to English-language full-text publications; CENTRAL was not searched, and citation tracking was not used as a formal supplementary retrieval step. Relevant evidence indexed elsewhere, reported in other languages, or available only in grey literature may therefore have been missed, and nine potentially eligible reports (9 of 135 assessed for full-text eligibility) could not be retrieved, which may have introduced availability bias. Recent systematic reviews and pooled analyses addressing radiation-induced lymphopenia or radiotherapy/immunochemotherapy toxicity were considered contextually but were not included in the primary synthesis because the present review focused on ALC-defined lymphopenia as a prognostic exposure among solid tumor patients treated with ICIs rather than radiotherapy-induced lymphopenia or lymphopenia reported primarily as treatment toxicity [14,15].

The review also prioritized conservative data-handling decisions that favor interpretive clarity and reduce analytic artifacts, including distinguishing direct ALC-defined lymphopenia from derived immune constructs and avoiding reconstruction of effect estimates from Kaplan-Meier curves. While this approach strengthens internal validity and helps preserve comparability of the primary exposure, it reduces inclusiveness and may leave some reported associations available only for contextual interpretation. It also reduced the number of studies contributing directly extractable HRs to the synthesis, and the proportion of studies contributing adjusted versus only qualitative or unadjusted information was not formally tabulated. In addition, formal evaluation of reporting biases and formal grading of certainty across outcomes were not performed, which limits the ability to make structured statements about confidence in the body of evidence.

This issue is particularly relevant for large non-binary ALC studies such as Goldschmidt et al. (2023), which was retained as contextual ALC evidence because it used quintile-based categorization rather than a fixed binary lymphopenia threshold. Although this classification reflected the prespecified exposure framework of the review, the direction of its findings was concordant with the main qualitative pattern, suggesting that inclusion of large ALC-categorization studies in the primary synthesis might have reinforced rather than weakened the overall signal.

Use of multiple risk-of-bias tools also introduced an additional layer of methodological heterogeneity. Although tool selection was based on the dominant analytical framing of each report, the boundary between direct exposed-versus-reference comparisons and broader prognostic factor analyses was not always clear-cut. This should be considered when interpreting study-level judgments across the evidence base.

Formal inter-rater agreement statistics were not calculated for risk-of-bias assessment. The absence of kappa values limits the ability to quantify initial reviewer agreement before consensus and should be considered when interpreting the reproducibility of study-level judgments.

Implications for Practice, Policy, and Future Research

Within the boundaries of the included evidence, ALC-defined lymphopenia appears to function as a prognostic correlate in some ICI-treated populations but does not show consistent prognostic meaning across all solid tumors and clinical contexts. Harmonized reporting of baseline and longitudinal hematologic parameters in immunotherapy trials and registries would improve comparability across studies and support more reliable prognostic evaluation. The observed heterogeneity suggests that lymphopenia is better interpreted as a marker of broader patient and disease context than as a standalone variable with uniform implications. These findings support cautious interpretation of ALC in clinical narratives and highlight its potential role as a covariate for risk adjustment and stratification in observational and translational studies, without implying that it should guide treatment selection.

For research reporting and synthesis, greater standardization is a clear priority. Future studies should explicitly distinguish baseline from on-treatment lymphopenia, prespecify clinically and biologically motivated measurement windows, and report the rationale for ALC thresholds and the units used. Approaches that model ALC as a continuous exposure, evaluate non-linear relationships, and report sensitivity to alternative cut-offs may provide a more informative representation of prognostic gradients than simple dichotomization [40]. For on-treatment lymphopenia, analytic strategies should be aligned with the underlying question and should address time-dependent confounding, for example, by using prespecified landmark analyses or time-varying models, with clear reporting of how early discontinuation and missing laboratory measurements were handled.

Future research should also aim to clarify whether and how peripheral lymphocyte counts relate to tumor-immune biology in ICI-treated solid tumors. Studies that integrate ALC with immune phenotyping, intratumoral immune measures, and established biomarkers may better delineate the biological and clinical meaning of lymphopenia [7,41,42]. Well-powered prospective cohorts with systematic covariate capture, as well as analyses of clinical trial datasets with standardized outcome assessment, are likely to be particularly informative. Where prognostic models are developed, robust methodological practices, external validation, and transparent reporting will be essential to mitigate overfitting and improve transportability. Taken together, these steps may reduce heterogeneity, support more reliable synthesis, and clarify the contexts in which lymphopenia provides clinically relevant prognostic information.

## Conclusions

Across 21 studies including diverse solid tumor populations treated with ICIs, several studies reported associations between lower ALC and less favorable survival outcomes, although findings were heterogeneous and context-dependent. The strength and consistency of these associations varied substantially across tumor types, ALC thresholds, timing of assessment, and analytical approaches, precluding a single generalizable prognostic interpretation.

Inference is limited by the predominance of retrospective observational designs, residual confounding, and variability in how lymphopenia was defined, timed, and analyzed. These constraints were reflected in risk-of-bias assessments, including concerns related to confounding, selective reporting, and limited sample size in some reports. Within this evidence base, ALC-defined lymphopenia is best interpreted as a pragmatic marker that may capture broader host and disease context rather than a uniform prognostic classifier across ICI-treated solid tumors. Current evidence does not establish whether lymphopenia predicts differential benefit from ICIs compared with other systemic therapies. Future studies using prospectively collected data or standardized trial datasets should distinguish baseline from on-treatment lymphopenia, prespecify clinically meaningful ALC assessment windows, and apply time-aware analytical approaches to clarify when lymphopenia provides reproducible prognostic information.

## Appendices

Supplementary material 1

The following database-specific strategies were used to operationalize the final search concepts for this review. Searches were run from database inception to 5 January 2026, with no start-date restriction.

**Table 3:** Reproducible electronic search strategies. Limits applied across databases during screening and eligibility assessment included human adult populations, solid tumors treated with ICIs, English-language full-text reports, and original clinical studies. Targeted supplementary searches combining the core lymphopenia/immunotherapy concepts with tumor-specific terms such as melanoma and lung cancer were also used when needed to improve retrieval. Citation tracking was not used as a formal supplementary search procedure.

Database	Search Strategy
PubMed (NCBI)	((("Immune Checkpoint Inhibitors"[Mesh]) OR "immune checkpoint inhibitor*"[Title/Abstract] OR immunotherapy[Title/Abstract] OR PD-1[Title/Abstract] OR PD-L1[Title/Abstract] OR CTLA-4[Title/Abstract] OR nivolumab[Title/Abstract] OR pembrolizumab[Title/Abstract] OR atezolizumab[Title/Abstract] OR ipilimumab[Title/Abstract]) AND (lymphopenia[Title/Abstract] OR lymphopaenia[Title/Abstract] OR "absolute lymphocyte count"[Title/Abstract] OR ALC[Title/Abstract]) AND (survival[Title/Abstract] OR prognosis[Title/Abstract] OR outcome*[Title/Abstract] OR "overall survival"[Title/Abstract] OR "progression-free survival"[Title/Abstract] OR PFS[Title/Abstract] OR OS[Title/Abstract])) NOT (animals[mh] NOT humans[mh])
EMBASE	(('immune checkpoint inhibitor'/exp OR 'immune checkpoint inhibitor*':ti,ab OR immunotherapy:ti,ab OR 'pd-1':ti,ab OR 'pd-l1':ti,ab OR 'ctla-4':ti,ab OR nivolumab:ti,ab OR pembrolizumab:ti,ab OR atezolizumab:ti,ab OR ipilimumab:ti,ab) AND (lymphopenia:ti,ab OR lymphopaenia:ti,ab OR 'absolute lymphocyte count':ti,ab OR alc:ti,ab) AND (survival:ti,ab OR prognosis:ti,ab OR outcome*:ti,ab OR 'overall survival':ti,ab OR 'progression-free survival':ti,ab OR pfs:ti,ab OR os:ti,ab)) NOT ([animals]/lim NOT [humans]/lim)
Scopus (Elsevier)	TITLE-ABS-KEY ( ("immune checkpoint inhibitor*" OR immunotherapy OR "PD-1" OR "PD-L1" OR "CTLA-4" OR nivolumab OR pembrolizumab OR atezolizumab OR ipilimumab) AND (lymphopenia OR lymphopaenia OR "absolute lymphocyte count" OR ALC) AND (survival OR prognosis OR outcome* OR "overall survival" OR "progression-free survival" OR PFS OR OS) ) AND NOT TITLE-ABS-KEY (animal* OR mouse OR murine)
Web of Science (Clarivate)	TS=((("immune checkpoint inhibitor*" OR immunotherapy OR "PD-1" OR "PD-L1" OR "CTLA-4" OR nivolumab OR pembrolizumab OR atezolizumab OR ipilimumab) AND (lymphopenia OR lymphopaenia OR "absolute lymphocyte count" OR ALC) AND (survival OR prognosis OR outcome* OR "overall survival" OR "progression-free survival" OR PFS OR OS))) NOT TS=(animal* OR mouse OR murine)

Supplementary material 2

**Table 4:** Direction of reported ALC contrasts in qualitative synthesis.

Study	Reported Contrast	Original Direction of Association	Common Prognostic Interpretation
Cho et al. (2019) [11]	Lymphopenia vs no lymphopenia	HR >1 indicates worse outcomes with lymphopenia	Directionally adverse for lymphopenia
Foerster et al. (2025) [32]	Higher ALC vs lower ALC	HR <1 indicates better outcomes with higher ALC	Directionally adverse for lower ALC / lymphopenia
Ho et al. (2018) [33]	Low pretreatment ALC vs higher ALC	HR >1 indicates worse outcomes with lower ALC	Directionally adverse for lymphopenia
Pike et al. (2019) [34]	Severe lymphopenia vs no severe lymphopenia	HR >1 indicates worse outcomes with severe lymphopenia	Directionally adverse for lymphopenia
Postow et al. (2020) [17]	Higher ALC vs lower ALC	HR <1 indicates better outcomes with higher ALC	Directionally adverse for lower ALC / lymphopenia
Takemura et al. (2024) [12]	Lymphopenia vs no lymphopenia	HR >1 indicates worse outcomes with lymphopenia	Directionally adverse for lymphopenia
Zhao et al. (2022) [25]	Lower ALC category vs higher ALC category	HR >1 indicates worse outcomes with lower ALC	Directionally adverse for lymphopenia
Lee et al. (2022) [30]	Highest vs lowest post-treatment ALC quartile	HR <1 indicates better outcomes with higher post-treatment ALC	Directionally adverse for lower ALC

Supplementary material 3

**Table 5:** Descriptive grouping of ALC thresholds used across included studies.

Study	Reported Contrast	Original Direction of Association	Common Prognostic Interpretation
Cho et al. (2019) [11]	Lymphopenia vs no lymphopenia	HR >1 indicates worse outcomes with lymphopenia	Directionally adverse for lymphopenia
Foerster et al. (2025) [32]	Higher ALC vs lower ALC	HR <1 indicates better outcomes with higher ALC	Directionally adverse for lower ALC / lymphopenia
Ho et al. (2018) [33]	Low pretreatment ALC vs higher ALC	HR >1 indicates worse outcomes with lower ALC	Directionally adverse for lymphopenia
Pike et al. (2019) [34]	Severe lymphopenia vs no severe lymphopenia	HR >1 indicates worse outcomes with severe lymphopenia	Directionally adverse for lymphopenia
Postow et al. (2020) [17]	Higher ALC vs lower ALC	HR <1 indicates better outcomes with higher ALC	Directionally adverse for lower ALC / lymphopenia
Takemura et al. (2024) [12]	Lymphopenia vs no lymphopenia	HR >1 indicates worse outcomes with lymphopenia	Directionally adverse for lymphopenia
Zhao et al. (2022) [25]	Lower ALC category vs higher ALC category	HR >1 indicates worse outcomes with lower ALC	Directionally adverse for lymphopenia
Lee et al. (2022) [30]	Highest vs lowest post-treatment ALC quartile	HR <1 indicates better outcomes with higher post-treatment ALC	Directionally adverse for lower ALC

Supplementary material 4

The analysis framework used for the structured qualitative synthesis specified the following elements before final full-text assessment and extraction: eligible populations were adults with solid tumors treated with PD-1, PD-L1, and/or CTLA-4 inhibitors; eligible exposures were ALC-defined lymphopenia measured before ICI initiation or after treatment initiation; the primary outcome was OS and the key secondary outcome was PFS, with TTP handled separately when PFS was not defined; direct ALC-defined lymphopenia was distinguished from derived leukocyte ratios and multivariable immune constructs; baseline and on-treatment lymphopenia were analyzed as distinct exposure timings; adjusted HRs were prioritized when available; studies were grouped by exposure directness, timing, tumor context, and analytical approach; contextual ALC or derived-construct studies were retained for directional interpretation but were not treated as equivalent to fixed binary ALC-defined lymphopenia; and no quantitative pooling was planned unless studies were sufficiently homogeneous in population, exposure timing, threshold definition, and outcome reporting.

No eligibility criteria, outcome definitions, or broad synthesis plans were changed after title and abstract screening began. The data extraction domains, risk-of-bias tool assignment rules, and four interpretive study categories were finalized before full-text assessment and extraction.

Supplementary material 5

**Table 6:** Decision algorithm for assigning risk-of-bias tools.

Decision Step	Rule Applied	Risk-of-Bias Tool Assigned
Step 1	Study primarily developed, validated, or evaluated a multivariable prognostic or prediction model	PROBAST
Step 2	Study explicitly compared outcomes between an ALC-defined lymphopenia group and a reference group, usually using a binary threshold	ROBINS-I, adapted for prognostic exposure comparisons
Step 3	Study evaluated ALC or related immune measures as prognostic factors without a dominant exposed-versus-reference comparison	QUIPS
Step 4	Study used derived leukocyte ratios, dynamic immune constructs, or non-binary ALC categorizations mainly for contextual interpretation	QUIPS or PROBAST according to analytical framing
Step 5	Borderline cases were discussed by two reviewers, with arbitration by a third reviewer when consensus was not reached	Final tool assigned by consensus

Supplementary material 6

**Table 7:** Domain-level risk-of-bias summary.

Study	Tool	Confounding / Prognostic Factor Measurement	Selection / Participation	Exposure / Predictor Classification	Missing Data / Attrition	Outcome Measurement	Analysis / Reporting	Overall Judgment
Cho et al. (2019) [11]	ROBINS-I	Moderate concern	Moderate concern	Low concern	Moderate concern	Low concern	Moderate concern	Moderate
Takemura et al. (2024) [12]	ROBINS-I	Moderate concern	Moderate concern	Low concern	Moderate concern	Low concern	Moderate concern	Moderate
Diehl et al. (2017) [16]	ROBINS-I	Moderate concern	Moderate concern	Moderate concern	Moderate concern	Low concern	Moderate concern	Moderate
Postow et al. (2020) [17]	ROBINS-I	Low concern	Low concern	Low concern	Low concern	Low concern	Low concern	Low
Park et al. (2020) [18]	QUIPS	Moderate concern	Moderate concern	Moderate concern	Moderate concern	Low concern	Moderate concern	Moderate
Karantanos et al. (2019) [26]	ROBINS-I	Serious concern	Serious concern	Moderate concern	Moderate concern	Low concern	Serious concern	Serious
Lee et al. (2022) [30]	QUIPS	Moderate concern	Moderate concern	Moderate concern	Moderate concern	Low concern	Moderate concern	Moderate
Goldschmidt et al. (2023) [27]	QUIPS	Moderate concern	Moderate concern	Moderate concern	Moderate concern	Low concern	Moderate concern	Moderate
Zhen et al. (2024) [22]	PROBAST	High concern	Moderate concern	Moderate concern	Moderate concern	Low concern	High concern	High
Foerster et al. (2025) [32]	ROBINS-I	Moderate concern	Moderate concern	Moderate concern	Moderate concern	Low concern	Moderate concern	Moderate
Tomsitz et al. (2022) [23]	QUIPS	Moderate concern	Moderate concern	Moderate concern	Moderate concern	Low concern	Moderate concern	Moderate
Ueda et al. (2023) [24]	PROBAST	High concern	Moderate concern	Moderate concern	Moderate concern	Low concern	High concern	High
Yin et al. (2022) [29]	ROBINS-I	Moderate concern	Moderate concern	Moderate concern	Moderate concern	Low concern	Moderate concern	Moderate
Zhao et al. (2022) [25]	ROBINS-I	Moderate concern	Moderate concern	Low concern	Moderate concern	Low concern	Moderate concern	Moderate
Ho et al. (2018) [33]	ROBINS-I	Moderate concern	Serious concern	Low concern	Moderate concern	Low concern	Moderate concern	Moderate
Pike et al. (2019) [34]	ROBINS-I	Moderate concern	Moderate concern	Moderate concern	Moderate concern	Low concern	Moderate concern	Moderate
Sun et al. (2017) [28]	ROBINS-I	Moderate concern	Moderate concern	Low concern	Moderate concern	Low concern	Moderate concern	Moderate
Conroy et al. (2024) [35]	ROBINS-I	Moderate concern	Moderate concern	Low concern	Moderate concern	Low concern	Moderate concern	Moderate
Du et al. (2023) [31]	PROBAST	High concern	Moderate concern	Moderate concern	Moderate concern	Low concern	High concern	High
Soyano et al. (2018) [36]	QUIPS	Moderate concern	Moderate concern	Moderate concern	Moderate concern	Low concern	Moderate concern	Moderate
Maymani et al. (2018) [37]	QUIPS	Moderate concern	Moderate concern	Moderate concern	Moderate concern	Low concern	Moderate concern	Moderate

Supplementary material 7

**Table 8:** Covariate adjustment domains and residual confounding concerns.

Study	Multivariable Survival Modeling Status	Covariate Domains Reported or Extracted for Adjustment	Residual Confounding Concern
Cho et al. (2019) [11]	Adjusted survival estimates reported	Clinical and treatment-related domains; radiotherapy-related factors	Residual confounding by disease burden, treatment line, and radiotherapy intensity remained possible
Takemura et al. (2024) [12]	Adjusted survival estimates reported	IMDC-related clinical domains and treatment context	Residual confounding by registry covariate completeness and disease burden remained possible
Diehl et al. (2017) [16]	Adjusted or model-based progression analysis reported	Clinical and treatment-related domains	Post-baseline timing and time-related bias remained possible
Postow et al. (2020) [17]	Trial-derived adjusted/model-based analysis reported	Trial and treatment-related domains	Residual confounding was lower than in retrospective cohorts but contrast direction required careful interpretation
Park et al. (2020) [18]	Adjusted PFS association reported but not fully extractable	Clinical and treatment-related domains incompletely extractable	Residual confounding and limited reporting remained possible
Karantanos et al. (2019) [26]	Primarily unadjusted analysis	Not applicable; no adequate multivariable survival adjustment extracted	Serious residual confounding concern because of very small sample and unadjusted analyses
Lee et al. (2022) [30]	Adjusted survival estimates reported	Clinical and treatment-related domains	Residual confounding by post-treatment measurement timing and disease trajectory remained possible
Goldschmidt et al. (2023) [27]	Large EHR-based adjusted/model-based analysis	Demographic, tumor, treatment, and laboratory domains as available in EHR	Residual confounding from missing EHR covariates and unmeasured performance status remained possible
Zhen et al. (2024) [22]	Prediction/model-based response analysis	Dynamic lymphocyte subset and treatment domains	High concern for overfitting and residual confounding
Foerster et al. (2025) [32]	Adjusted or exploratory model-based estimates reported	Clinical, metastatic pattern, and laboratory domains	Exploratory cut-off selection and residual confounding remained possible
Tomsitz et al. (2022) [23]	Unadjusted or incompletely adjusted ALC survival contrasts	Limited extractable covariate adjustment for ALC-defined contrasts	Residual confounding by disease course and treatment exposure remained possible
Ueda et al. (2023) [24]	Prognostic model analysis	Clinical and treatment-related model domains	High overfitting concern because of small derivation cohort
Yin et al. (2022) [29]	Adjusted/model-based PFS analysis reported	Clinical and treatment-related domains	Residual confounding by combined treatment setting remained possible
Zhao et al. (2022) [25]	Adjusted/model-based OS analysis reported	Clinical and tumor-related domains	Residual confounding in a restricted disease setting remained possible
Ho et al. (2018) [33]	Adjusted or model-based PFS analysis reported	Clinical and treatment-related domains	Residual confounding and small-sample imprecision remained possible
Pike et al. (2019) [34]	Adjusted survival estimates reported	Clinical, disease, radiotherapy, and treatment-related domains	Residual confounding by indication and disease burden remained possible
Sun et al. (2017) [28]	Adjusted/model-based survival analysis reported	Trial population and clinical domains	Residual confounding and phase I population selection remained possible
Conroy et al. (2024) [35]	Adjusted contrasts incompletely reported	Clinical and treatment-related domains incompletely extractable	Residual confounding and incomplete adjusted reporting remained possible
Du et al. (2023) [31]	Prognostic model analysis	Dynamic immune and clinical domains	High overfitting and residual confounding concern
Soyano et al. (2018) [36]	Derived ratio survival analysis	Clinical and treatment-related domains	Indirect exposure construct and residual confounding remained possible
Maymani et al. (2018) [37]	Primarily univariate or limited multivariable subgroup analysis	Limited extractable adjustment domains	Small sample and residual confounding remained possible

Supplementary material 8

**Table 9:** Narrative certainty assessment by evidence domain.

Evidence Domain	Directional Pattern	Main Downgrading Considerations	Narrative Certainty
Baseline ALC-defined lymphopenia and OS	Frequently adverse, but not uniform across studies	Retrospective designs, residual confounding, threshold heterogeneity, variable adjustment	Low
Baseline or early-treatment ALC-defined lymphopenia and PFS/TTP	Generally adverse in several settings	Heterogeneous outcome definitions, timing differences, limited extractable adjusted estimates	Low to very low
On-treatment lymphopenia and survival outcomes	Suggestive adverse pattern in some studies	Time-dependent confounding, reverse causation, landmark heterogeneity, incomplete adjustment	Very low
Contextual ALC categorizations	Directionally consistent with lower ALC being less favorable	Non-binary thresholds, quantile-based coding, limited direct comparability	Low to very low
Derived immune constructs	Broadly compatible but indirect	Not equivalent to direct ALC-defined lymphopenia, ratio-based exposure mixing	Very low

Supplementary material 9

**Table 10:** Harmonized qualitative direction of ALC-related associations.

Study	Main Reported ALC Contrast	Outcome(s) Informing Direction	Original Coding Direction	Harmonized Qualitative Direction
Cho et al. (2019) [11]	Lymphopenia vs no lymphopenia	OS, PFS	HR >1 indicates worse outcomes with lymphopenia	Adverse for lymphopenia
Takemura et al. (2024) [12]	Baseline lymphopenia vs no lymphopenia	OS, ORR, TTNT	HR >1 indicates worse outcomes with lymphopenia	Adverse for lymphopenia
Diehl et al. (2017) [16]	Lower ALC / lymphopenia vs reference	TTP	Lower ALC associated with shorter TTP	Adverse for lower ALC
Postow et al. (2020) [17]	Higher ALC vs lower ALC	OS	HR <1 indicates better outcomes with higher ALC	Adverse for lower ALC
Park et al. (2020) [18]	Week 6 low ALC vs higher ALC	PFS, OS, ORR	Lower week 6 ALC associated with worse PFS	Adverse for lower ALC
Karantanos et al. (2019) [26]	ALC quartiles / week 6 ALC	OS, time on treatment	Exploratory categorization	Suggestive adverse direction for lower ALC, lower certainty
Lee et al. (2022) [30]	Highest vs lowest post-treatment ALC quartile	PFS, OS, ORR	HR <1 indicates better outcomes with higher ALC	Adverse for lower ALC
Goldschmidt et al. (2023) [27]	Lowest vs highest ALC quintiles	OS, TTD, TTNT	Lower quintiles associated with worse outcomes	Adverse for lower ALC categories
Zhen et al. (2024) [22]	Dynamic post-cycle to baseline lymphocyte ratios	Response	Dynamic construct, not binary ALC	Indirect; not directly harmonized
Foerster et al. (2025) [32]	Higher ALC vs lower ALC	PFS, OS, ORR	HR <1 indicates better outcomes with higher ALC	Adverse for lower ALC
Tomsitz et al. (2022) [23]	Developed lymphopenia vs no developed lymphopenia	PFS, OS	Shorter survival with developed lymphopenia	Adverse for on-treatment lymphopenia
Ueda et al. (2023) [24]	Low pretreatment ALC vs higher ALC	PFS, OS, ORR	HR >1 indicates worse outcomes with low ALC	Adverse for lower ALC
Yin et al. (2022) [29]	Low ALC / treatment-related lymphopenia	PFS, ORR, DCR	Lower ALC associated with worse PFS	Adverse for lymphopenia
Zhao et al. (2022) [25]	Lower ALC category vs higher ALC category	OS	HR >1 indicates worse outcomes with lower ALC	Adverse for lower ALC
Ho et al. (2018) [33]	Low pretreatment ALC vs higher ALC	PFS, ORR, DCR	HR >1 indicates worse outcomes with low ALC	Adverse for lower ALC
Pike et al. (2019) [34]	Severe lymphopenia vs no severe lymphopenia	OS	HR >1 indicates worse outcomes with severe lymphopenia	Adverse for severe lymphopenia
Sun et al. (2017) [28]	Baseline lymphopenia vs no lymphopenia	OS, ORR, DCR	Near-null baseline OS association	No clear adverse association
Conroy et al. (2024) [35]	Baseline and 3-month lymphopenia	OS, PFS	Adverse 3-month association reported	Adverse for on-treatment lymphopenia, incompletely reported
Du et al. (2023) [31]	Dynamic lymphocyte-change construct	PFS, OS, ORR	Dynamic construct, not binary ALC	Indirect; not directly harmonized
Soyano et al. (2018) [36]	ANC:ALC ratio	OS, PFS, irAEs	Derived ratio, not direct ALC	Indirect; compatible but not equivalent
Maymani et al. (2018) [37]	NLR >6 vs lower NLR	OS, PFS	Derived ratio, not direct ALC	Indirect; compatible but not equivalent

Supplementary material 10

**Table 11:** ALC thresholds and timing windows across included studies.

Study	ALC Threshold / Exposure Definition	Timing Window	Harmonization Comment
Cho et al. (2019) [11]	ALC <1000 cells/mm³	Baseline / early treatment	Direct binary ALC-defined lymphopenia
Takemura et al. (2024) [12]	Baseline ALC <1000 cells/µL	Baseline	Direct binary ALC-defined lymphopenia
Diehl et al. (2017) [16]	ALC <1000 cells/µL	Baseline and 3 months	Direct ALC-defined timing comparison
Postow et al. (2020) [17]	ALC at 1.0 × 10⁹/L	Baseline and week 6	Inverse contrast; higher ALC reported
Park et al. (2020) [18]	Week 6 low ALC <770 cells/µL	Week 6	Early on-treatment landmark
Karantanos et al. (2019) [26]	Baseline quartiles and week 6 ALC	Baseline and week 6	Exploratory categorization
Lee et al. (2022) [30]	Post-treatment ALC quartiles	3 to 4 weeks	Non-binary post-treatment categorization
Goldschmidt et al. (2023) [27]	Lowest vs highest ALC quintiles by tumor type	Baseline	Non-binary EHR categorization
Zhen et al. (2024) [22]	Ratio of post-cycle to baseline counts	After treatment cycles	Dynamic ratio construct
Foerster et al. (2025) [32]	Baseline ALC dichotomized at 1.61 × 10³/µL	Baseline	Higher-than-usual threshold; inverse coding
Tomsitz et al. (2022) [23]	Lymphopenia <1000 cells/µL at any time	Any time during therapy	On-treatment occurrence, not fixed landmark
Ueda et al. (2023) [24]	Pretreatment low ALC <1289 cells/µL	Pretreatment	Higher-than-usual threshold
Yin et al. (2022) [29]	ALC <0.50 × 10⁹/L	Baseline / on-treatment	Severe lymphopenia threshold
Zhao et al. (2022) [25]	Baseline ALC ≤625 cells/µL	Baseline	Lower threshold
Ho et al. (2018) [33]	Pretreatment ALC <600 cells/µL	Pretreatment	Lower threshold
Pike et al. (2019) [34]	Severe lymphopenia: ALC <500 cells/µL	Start of treatment	Severe baseline lymphopenia
Sun et al. (2017) [28]	Baseline ALC <1 G/L	Baseline	Direct binary ALC-defined lymphopenia
Conroy et al. (2024) [35]	ALC <1.0 × 10⁹/L	Baseline and 3 months	Baseline and on-treatment assessment
Du et al. (2023) [31]	Decreased lymphocyte counts after 12 weeks	12 weeks	Dynamic post-baseline construct
Soyano et al. (2018) [36]	Baseline ANC:ALC ratio ≥5.9	Baseline	Derived leukocyte ratio
Maymani et al. (2018) [37]	Baseline NLR >6	Baseline	Derived leukocyte ratio

Supplementary material 11

**Table 12:** Reproducibility elements and location in the manuscript.

Reproducibility Element	Where Reported	Additional Clarification
Databases searched and final search date	Methods; Supplementary material 1	PubMed, EMBASE, Scopus, and Web of Science searched through 5 January 2026
Exact database-specific search strategies	Supplementary material 1	Boolean strings and database-specific syntax reported for all four databases
Eligibility criteria	Methods, Eligibility criteria	Population, exposure, comparator, outcomes, study designs, language/full-text limits, and exclusion criteria specified
Study selection process	Methods, Selection process; Figure 1	Two independent reviewers screened records and full texts; disagreements were resolved by consensus or third-reviewer arbitration
Data extraction	Methods, Data collection process and data items	Standardized extraction domains and prioritization rules for effect estimates reported
Risk-of-bias assessment	Methods, Risk of bias assessment; Table 2 and Supplementary material 6	Tool allocation rules and domain-level judgments reported
Handling of heterogeneous lymphopenia definitions	Methods, Effect measures and synthesis methods; Supplementary materials 3, 9, and 10	Baseline and on-treatment lymphopenia treated separately; thresholds and timing windows summarized descriptively
Outcome handling and synthesis	Methods, Effect measures and synthesis methods; Results; Supplementary material 12	OS and PFS prioritized; TTP and secondary outcomes described separately; qualitative synthesis used because pooling was not considered defensible
Protocol or analysis plan	Methods; Supplementary material 4	The protocol was not prospectively registered; core elements of the a priori analysis framework are reported for transparency

Supplementary material 12

**Table 13:** Feasibility assessment for candidate quantitative synthesis.

Candidate Quantitative Synthesis	Assessment	Main Reason Pooling Was Not Performed
Baseline lymphopenia and OS	Considered but not pooled	Studies differed in tumor type, ICI regimen, ALC threshold, effect-estimate direction, and adjustment level
Baseline lymphopenia and PFS	Considered but not pooled	Fewer directly comparable estimates; PFS definitions and imaging/assessment schedules varied across retrospective cohorts
On-treatment lymphopenia and OS/PFS	Considered but not pooled	Timing windows varied substantially, including week 6, 3 months, any-time lymphopenia, and dynamic post-baseline constructs
ALC <1000 cells/µL subgroup	Considered but not pooled	Threshold similarity did not overcome differences in tumor setting, exposure timing, outcome definition, and model adjustment
Tumor-specific subgroup meta-analysis	Considered but not pooled	Within-tumor strata contained too few sufficiently comparable studies for reliable random-effects or Hartung-Knapp estimation
Meta-regression by threshold or timing	Considered but not performed	Number of comparable studies was too small relative to the number of candidate modifiers
Publication-bias testing	Not performed	No pooled meta-analysis or sufficiently homogeneous study set was available for funnel plot or regression-based testing
